# Environment‐induced changes in reproductive strategies and their transgenerational effects in the three‐spined stickleback

**DOI:** 10.1002/ece3.7052

**Published:** 2020-12-09

**Authors:** Náyade Álvarez‐Quintero, Alberto Velando, Jose C. Noguera, Sin‐Yeon Kim

**Affiliations:** ^1^ Grupo Ecoloxía Animal (Lab 97) Torre CACTI Centro de Investigación Mariña Universidade de Vigo Vigo Spain

**Keywords:** *Gasterosteus aculeatus*, photoperiod, reproduction, transgenerational effects

## Abstract

An organism may increase its fitness by changing its reproductive strategies in response to environmental cues, but the possible consequences of those changes for the next generation have rarely been explored. By using an experiment on the three‐spined stickleback (*Gasterosteus aculeatus*), we studied how changes in the onset of breeding photoperiod (early versus late) affect reproductive strategies of males and females, and life histories of their offspring. We also explored whether telomeres are involved in the within‐ and transgenerational effects. In response to the late onset of breeding photoperiod, females reduced their investment in the early clutches, but males increased their investment in sexual signals. Costs of increased reproductive investment in terms of telomere loss were evident only in the late females. The environmentally induced changes in reproductive strategies affected offspring growth and survival. Most notably, offspring growth rate was the fastest when both parents experienced a delayed (i.e., late) breeding photoperiod, and survival rate was the highest when both parents experienced an advanced (i.e., early) breeding photoperiod. There was no evidence of transgenerational effects on offspring telomere length despite positive parents–offspring relationships in this trait. Our results highlight that environmental changes may impact more than one generation by altering reproductive strategies of seasonal breeders with consequences for offspring viability.

## Graphical abstract

By using an experiment on the three‐spined stickleback, we studied how changes in the onset of breeding photoperiod (early versus late) affect reproductive strategies of breeders and life histories of their offspring. In response to the late onset of breeding photoperiod, females reduced their investment in the early clutches, but males increased their investment in sexual signals. The environmentally induced changes in reproductive strategies affected offspring growth and survival.

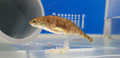

## 1 INTRODUCTION

Reproductive traits are among the most flexible phenotypic traits that change in response to environmental conditions or cues (Baker et al., 2015; Foster et al., 2015). In seasonal environments, for example, animals optimally adjust the timing of reproduction in response to temperature to match with the period of maximum food abundance et al (McNamara et al., 2011; Visser & Gienapp, 2019). Since reproduction is energetically costly and trades off against survival and future reproduction, iteroparous breeders adjust their investment in current reproduction according to their body condition and food availability (McNamara & Houston, 1996; Poizat et al., 1999; Stearns, 1989). The consequences of changes in the timing and intensity of reproductive activities in response to environmental conditions or cues have been studied by following fitness‐related traits of breeders (Thomas et al., 2001; Visser et al., 2004). Although these changes in reproductive strategies of breeders are likely to have a strong impact on offspring phenotype, their possible consequences for the next generation have rarely been explored.

When an environmental cue indicates that conditions are inappropriate for reproduction, animals may delay the onset of reproduction, reduce their investment in early reproduction or make more subtle changes in their reproductive strategies (Koons et al., 2008; Tuljapurkar, 1990). The consequences of these changes for offspring phenotype are not easy to predict but may depend on the responses of breeders at the time of reproduction (Verhulst & Nilsson, 2008; Wilson & Nussey, 2010). Breeders that delay reproductive activities may produce more viable offspring than early breeders if they invest more in self‐maintenance by reducing costs of early reproduction and consequently attain a better condition at the time of reproduction (Kulaszewicz et al., 2017; Metcalfe & Monaghan, 2013). The opposite scenario is also possible if breeders delay their physiological preparation for reproduction in response to environmental cues (McNamara & Houston, 1996). Before reproduction, animals develop gonads and secondary sexual traits (Hau et al., 2017; Sokolowska & Kulczykowska, 2006), accumulate macronutrients and micronutrients required for reproduction (Schneider, 2004) and restructure their internal organs (Jacobs et al., 2011; Madsen et al., 2015; Speakman, 2008). Breeders that delay these preparation processes may produce less viable offspring due to reduced maternal resource allocation (Tökölyi et al., 2012). The consequences of changes in the timing and intensity of reproductive activities for offspring may be more complex if male and female breeders respond differently to changes in environmental cues (Ball & Ketterson, 2008).

In seasonal environments, an important environmental cue that affects both male and female reproductive activities is photoperiod. In different taxa, it has been shown that a long‐day photoperiod often triggers male investment in sexual ornaments and courtship and territorial behaviors (Borg, 1982; Sharp, 2005; Walton et al., 2011). However, sexual signals are often costly to produce and their expression and maintenance may divert important resources away from other functions related to the maintenance of the soma and gonads (Simmons et al., 2017; Somjee et al., 2018). Indeed, several studies have shown that males investing more heavily in carotenoid‐based sexual signals may be exposed to increased oxidative stress levels (Blount et al., 2001, 2003; Kim & Velando, 2019) and experience a faster rate of aging (Jennions et al., 2001; Kim & Velando, 2016). In females, long‐day photoperiods often promote sexual maturity and gonadal development (Zutshi & Singh, 2020). This process may be especially important in oviparous species as females need a physiological preparatory period to accumulate enough energetic and antioxidant resources for egg formation (Fox & Czesak, 2000; Glazier, 1999; Jönsson, 1997). Hence, changes in the timing of reproductive photoperiod may induce sex‐specific changes in seasonal reproductive patterns and aging and potentially influence offspring phenotype (Ball & Ketterson, 2008; Caro et al., 2009).

Changes in reproductive strategies may influence offspring phenotype via different physiological and genetic mechanisms affecting gamete quality. Evidence indicates that heavy investment in sexual signalling may not only deplete resources needed to somatic maintenance but also those protecting and maintaining germline tissues (Blount et al., 2001; Tomášek et al., 2017). Male ejaculate, for instance, is more than simply spermatozoa as it often contains important amounts of proteins and antioxidants compounds (e.g., carotenoids and vitamins) that play a key function in protecting the vulnerable DNA of the spermatozoa from oxidative damage (Agarwal et al., 2012; Cabrita et al., 2014; Velando et al., 2008; Walczak‐Jedrzejowska et al., 2013). Similarly, offspring viability is influenced by egg size and composition (e.g., nutrients, hormones or antioxidants; Brown et al., 2014; Giesing et al., 2011; Palace & Werner, 2006), which are strongly influenced by the capacity of females to accumulate different resources before reproduction takes place (Elkin & Reid, 2005; Garrido et al., 2007). The timing of reproduction may affect germline integrity and egg composition, thereby inducing transgenerational effects on offspring viability (Monaghan & Metcalfe, 2019), although the mechanisms that mediate such changes are still not well understood.

One proximate mechanism that underlies both the within‐ and transgenerational effects of environmentally induced changes in reproductive activities may be found in the telomeres (Chatelain et al., 2020; Sudyka, 2019). Telomeres are noncoding nucleotide sequences that cap the ends of eukaryotic chromosomes and play an important role in chromosome protection (Blackburn, 1991; O'sullivan & Karlseder, 2010). In the majority of somatic tissues, telomeres decrease with age, and individuals with shorter telomeres have increased risk of disease and reduced longevity (Whittemore et al., 2019; Young, 2018). The exposure to free radicals and deficiency of antioxidant defenses can accelerate the rate of telomere loss (Pineda‐Pampliega et al., 2020; Reichert & Stier, 2017). Telomeres, however, can be restored by the action of the telomerase enzyme. The activity of this ribonucleoprotein, which is one of the most important telomere repair mechanisms in vertebrates, is extremely low in most somatic tissues in birds and mammals. However, fish, reptiles, and amphibians exhibit indeterminate somatic growth and express telomerase during their entire life (Olsson et al., 2018). Although the heritability of telomere length varies between species (Armanios & Blackburn, 2012; Olsson et al., 2018), the parental influence on offspring telomere length and dynamics is thought to be a key mechanism determining offspring life‐history trajectories (Monaghan, 2010).

**Figure 1:**
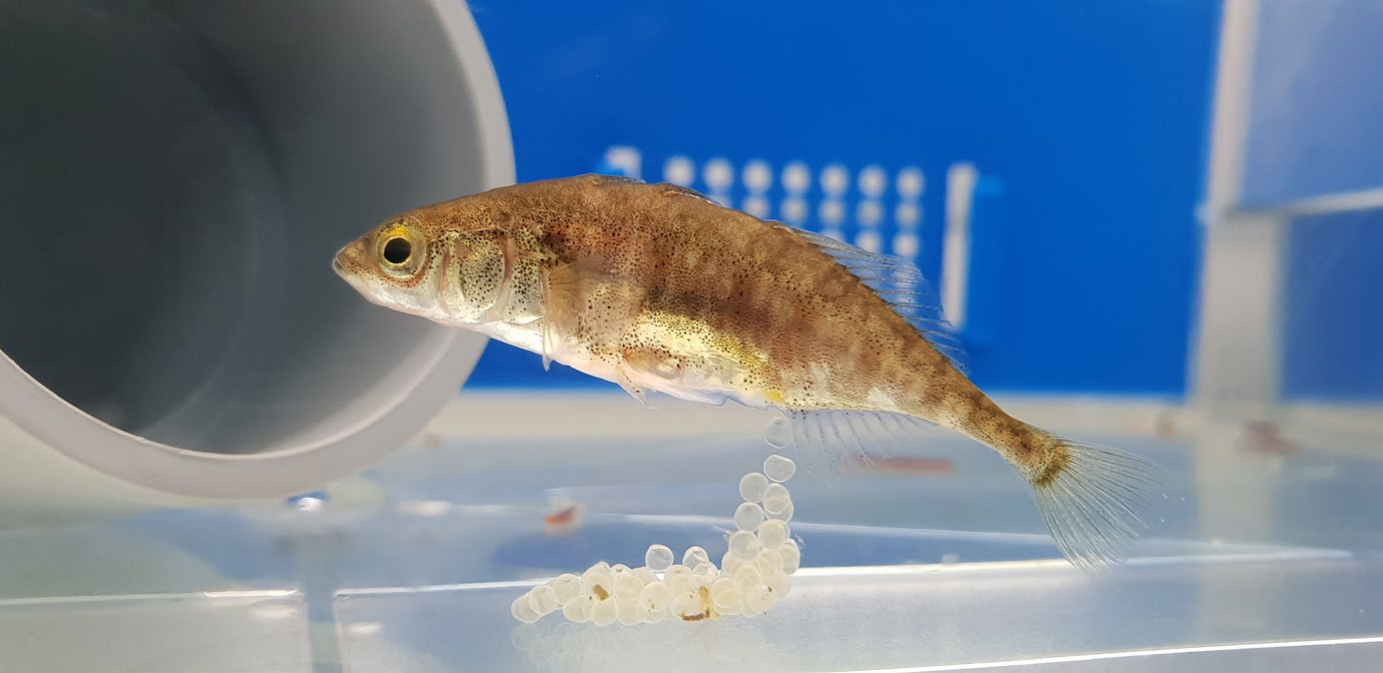
Photograph of a spawning female three‐spined stickleback (taken by N. A.‐Q. using a Samsung Galaxy S9+ digital camera)

Here, we investigate whether changes in the onset of the reproductive photoperiod affect male and female reproductive strategies and whether these have any carry‐over effect on offspring life‐history traits and telomere dynamics. For this, we carried out an experimental study on the three‐spined stickleback (*Gasterosteus aculeatus*) at the southern edge of its European range, where it reproduces repeatedly throughout a single relatively long breeding season. The three‐spined stickleback shows a marked variation in reproductive seasonality (Ishikawa & Kitano, 2020) and usually mature under a long‐day photoperiod (Borg et al., 2004; Noreikiene et al., 2017). Photoperiod acts as a cue that predicts the forthcoming of favorable conditions (e.g., the maximal food abundance) for breeding sticklebacks. Under a long‐day photoperiod, male sticklebacks sexually mature, expressing red nuptial coloration and establishing a nesting territory (Borg, 1982; Walton et al., 2011). In female sticklebacks, the production of eggs represents the major energetic cost of reproduction (Wootton & Evans, 1976), and both gonadal and somatic growth are sustained in parallel during the prebreeding period (Wootton et al., 1980; Figure 1). In males, although the production of gonads may impose a significant energetic investment, courtship and parental care involve the higher energetic costs (Huntingford et al., 2001; Smith & Wootton, 1999).

For this, we manipulated the onset of reproductive photoperiod to induce changes in reproductive strategies and tested their within‐ and transgenerational effects by using three‐spined sticklebacks from a wild population. We created two experimental groups of fish in which the onset of reproductive photoperiod was either advanced or delayed (hereafter, early and late group, respectively) and observed how this manipulation influences male and female reproductive investment (i.e., date of nest building, sexual coloration, clutch size, etc.). Then we crossed the experimental male and female breeders in a factorial 2 × 2 design to disentangle the maternal, paternal and combined influences of the experimental treatment on offspring growth and survival. In addition, we studied a potential mechanism underlying the transgenerational effects by exploring telomere dynamics (and telomerase activity) of the two generations.

## 2 MATERIAL AND METHODS

### 2.1 Study population and experimental design

A total of 100 sexually immature three‐spined sticklebacks were captured in the Rio Ulla, Spain, in October 2016. Fish were acclimated to laboratory conditions and individually housed in 8‐L tanks under a constant winter photoperiod [light:dark (LD) 9h:15h] and water temperature controlled to conform to thermal conditions in their natural habitat (initially 15°C, then gradually decreased to 12°C in winter). Fish were fed to satiation three times per week with a commercial diet (Gemma Micro, Skretting, Norway).

In February 2017, 60 fish of unknown sex were randomly assigned to either an “early” (*N* = 30) or “late” (*N* = 30) breeding season photoperiod treatment group. Fish assigned to the same experimental treatment were allocated to one of two independent and identical aquaria systems (30 tanks per system) where they were individually housed in 8‐L tanks. In each aquaria system, water was continuously filtered, aerated and temperature‐controlled following the water temperature in nature (initially 12°C, then gradually increased to 20°C in summer) by the combined flow‐through function. Since sexual maturation is stimulated by long photoperiod in this species (Borg, 1982), we manipulated the photoperiod regimes to control the onset of reproductive activity. The experimental photoperiod was simulated by programmed illumination. In the early group, fish were kept under the breeding season photoperiod (LD 15h:9h) from early February onwards, whereas in the late group, the beginning of the breeding season photoperiod was postponed 1 month, until early March. Thus, the early fish started receiving visual signals indicating the upcoming breeding season 1 month before the late group. Afterward, both groups were maintained under the breeding season photoperiod until they were sacrificed in June (see below). Before the experimental manipulation, all fish were measured and weighed (standard length and body mass), and a small sample of tissue was taken from the dorsal fin and stored at −80°C for telomere analysis (see below). There were no initial differences between groups and sexes in standard length (LM; photoperiod treatment: *F*_1,51_ = 0.402, *p* = .528; sex: *F*_1,51_ = 0.585, *p* = .448; treatment × sex: *F*_1,50_ = 0.062, *p* = .804) and body mass (LM; photoperiod treatment: *F*_1,51_ = 0.535, *p* = .467; sex: F_1,51_ = 0.755, *p* = .389; treatment × sex: *F*_1,50_ = 0.274, *p* = .603).

### 2.2 Measurements of male and female reproductive traits

Matured males that began to express red nuptial coloration were provided with sand and polyester threads as nest materials and were shown a nonexperimental gravid female from the same population, enclosed in a transparent glass, for 5 min twice a week to prompt breeding activities since early March for the early group and early April for the late group. The males were then monitored daily to record the date of nest building and photographed on their lateral side in late April and late May using a digital camera (Nikon D90, Nikon Corp.) under standardized conditions. We then calculated the relative size of the red area in relation to the total lateral body area as a proxy of male investment in sexual coloration from the digital images by using IMAGE analysis software (analySIS FIVE, Olympus) and following a previously described protocol (Kim et al., 2016). We repeatedly photographed the males because red coloration dynamically changes throughout the season, and different timing of reproductive activities might produce different temporal patterns in coloration between the two experimental groups.

The experimental females were also monitored daily to register all spawning events, which were evident from changes in the abdomen size. A total of 25 females (15 early and 10 late) spawned between March and June 2017, producing 151 clutches (94 early and 57 late). Whenever a female became fully gravid, the egg clutch was stripped by applying gentle pressure to the abdomen under a light benzocaine anesthetic. The clutch was gently spread on a piece of blotting paper using a fine painting brush to determine the clutch size (i.e., total number of eggs). On 31 occasions out of 151 (18 early and 13 late), gravid females spawned in their tanks before we could strip and count the eggs, but the spawning events were recorded. The date of the first spawning, the total number of spawning events and clutch size were used for data analyses.

All experimental males and females that survived until early June (*N = *54) were sacrificed with an overdose of benzocaine anesthetic and their standard length measured (to the nearest 1 mm) with a ruler. Tissue samples from the dorsal fin and skeletal muscle were collected and kept at −80°C for posterior laboratory analyses of telomere length and *TERT* expression (a catalytic subunit of the telomerase enzyme; see below).

### 2.3 Measurements of offspring traits

To assess the transgenerational effects of the photoperiod treatment, we repeatedly used all experimental fish (25 females and 29 males) for breeding and obtained 42 full‐sibling F1 families during April‐June 2017. Among the experimental fish, 20 females (12 early and 8 late) and 22 males (11 early and 11 late) succeed to breed up to three times, each time with a different mate. A total of 6 females and 3 males crossed three times, 10 females and 15 males crossed twice, and 4 females and 4 males crossed only once. These crosses resulted in four different family groups: early mother × early father (*N = *11 families), early mother × late father (*N* = 12), late mother × early father (*N* = 10), and late mother × late father (*N* = 9). There were no differences in the date of the crosses between groups (LM; mother treatment: *F*_1,38_ = 0.034, *p* = .854; father treatment: *F*_1,38_ = 0.066, *p* = .798; mother × father treatment: *F*_1,38_ = 0.048, *p* = .828).

In each breeding attempt, a fully gravid female was introduced into a male's tank and allowed to spawn naturally in the male's nest. After fertilization, the female was returned to its tank. The whole clutch was collected from the nest 2 hr after fertilization. A subsample of 5 eggs was carefully collected from the clutch and gently spread on a piece of blotting paper using a fine paint brush then photographed using a digital camera (Nikon D90, Nikon Corp.) under standardized conditions within a black box containing LED illumination. The average egg size of each clutch was calculated from the digital image using the ImageJ software (Rasband, 1997). The rest of the clutch was incubated inside a plastic cup with a mosquito net on the bottom in a 100‐L tank that housed all the clutches following the standard egg husbandry protocol described in (Barber & Arnott, 2000). The day before the expected hatching date (i.e., 7th day of incubation), each clutch was transferred from the incubation tank to an individual hatching tank with a sponge filter (one clutch per hatching tank). The clutches were then monitored to record hatching success. Two clutches (an early mother × early father and a late mother × early father) did not develop properly due to a fungi infection and so were not taken into account in the analyses (see below). Fish larvae (mean ± *SE* number of larvae per full‐sib family: 27.25 ± 1.63) were fed to satiation twice a day (morning and afternoon) on a progressive diet of newly hatched *Artemia* until age 40 days, and a commercial pelleted diet from age 15 days onwards (Gemma Micro, Skretting, Norway). At age 4 days, when the yolk was completely absorbed, 5 larvae from the 40 full‐sib families that hatched were randomly selected and sacrificed with an overdose of benzocaine anesthetic and immediately stored at −80°C for posterior telomere length analyses (see below). The survival of all F1 fish was monitored daily until age 40 days by visual inspection of the tanks and the date of death recorded.

At age 40 days, a subsample of juvenile fish from each experimental family (range: 2–12 individuals, depending on the number of fish available) was randomly selected, weighed and measured (body mass and standard length) to assess the effect of maternal and paternal photoperiod on offspring growth. All larvae of three families (a late mother × late father and two late mother × early father) died before age 40 days, and so we measured mass and length of 292 juveniles from the remaining 37 families.

### 2.4 Telomere length analysis

DNA was extracted with commercial kits (Quick‐DNA Miniprep Plus Kit; Zymo Corp) from dorsal fin tissues of the F0 individuals, which were sampled before and after the photoperiod manipulation, and the whole F1 larvae sampled at age 4 days. The relative telomere length of each sample was then measured using real‐time quantitative PCR (RT‐qPCR) following a method developed by Cawthon (2002) and validated in the three‐spined stickleback (Kim et al., 2019). Details of primer sequences, amplicon sizes, melting temperature and efficiency are provided in the Supporting Information (Table S1; see also Kim et al., 2019). In the assay, the relative telomere length of each sample was measured by determining the ratio (T/S) of telomere repeat copy number (T) to single control gene copy number (S), relative to a reference sample. The three‐spined stickleback glyceraldehyde‐3‐phosphate dehydrogenase gene (GAPDH) was used as the single control gene (Kim et al., 2019). The telomere and GAPDH reactions were carried out on separate plates and the efficiency of each amplicon was estimated from the slopes of the amplification curves for each qPCR and averaged for each amplicon using LinRegPCR software (Ruijter et al., 2009). In all cases, the reactions' efficiencies were within an acceptable range (see Table S1). All samples were run in triplicate, and quantification cycle (Cq) values were used to calculate the relative telomere length (T/S ratio) relative to the reference sample, controlling for amplicon efficiency as described in Pfaffl (2001). The reference sample was also used to calculate both the within‐ and among‐plate coefficient of variation (CV). Mean intra‐assay and inter‐assay CV of the T/S ratios were 0.79 and 0.37 for TEL and 0.14 and 0.15 for GAPDH, respectively (see also Table S1).

### 2.5 *TERT* expression analysis

Telomere length regulation is a dynamic process, and one of the main mechanisms of telomere restoration is through the ribonucleoprotein enzyme telomerase (Wu et al., 2017). Gene expression profiles of the telomerase reverse transcriptase (TERT) have been shown to plays a key role in telomerase activity and telomere restoration (Cairney & Keith, 2008). For this study, the expression of the *TERT* gene was estimated based on relative quantification of mRNA transcripts by RT‐qPCR using StepOnePlus Real‐Time PCR Systems (Applied Biosystems, Forest City, CA). The skeletal muscle samples from the 54 adult fish (i.e., F0) sacrificed in June were embedded in 200 µl of RiboZol (Amresco) and homogenized using RNase‐free pellet pestles (Sigma‐Aldrich) until they were disaggregated. A volume of 800 µl RiboZol was added to each homogenized sample to obtain a final volume of 1 ml then total RNA was isolated following the RiboZol manufacturer's instructions. Samples were treated with DNase I to remove any contaminating DNA and the RNAs purified using the DNA‐Free RNA kit (Zymo Research).

For RT‐qPCR, the concentration of all the RNA samples was quantified using a Synergy HT (Biotek). First‐strand cDNAs were synthesized with qScript cDNA synthesis Kit (Quanta Biosciences) using 500 ng of total RNA. The cDNA was stored at −80°C until qPCR analysis. Gene‐specific primers were designed based on sequence information from the three‐spined stickleback genome assembly (www.ensembl.org/Gasterosteus_aculeatus/) and synthesized (Sigma‐Aldrich Quimica). The efficiency of gene‐specific primers was checked to ensure similar values in amplification. Details of primer sequences, amplicon sizes, melting temperature and efficiency are provided in the Supporting Information (Table S1). Elongation factor 1 alpha gene (*EF1α*) was used as a reference gene. The level of expression was measured in a 20 µl reaction volume, containing 0.8 µl of each primer (10 µM), 10 µl of Luminaris Color HiGreen qPCR Master Mix, high ROX (Thermo Fisher Scientific) and 3 µl of cDNA for gene expression of *TERT* or 1 µl of cDNA for *EF1α*. The cycling condition was set to 95°C for 10 min, followed by 40 cycles of 95°C for 15 s and 59°C for 1 min. All reactions were performed in triplicate and the averaged values were used for data analysis; the intra‐ and interassay CV for *TERT* and *EF1α* were 0.30/0.06 and 0.31/0.02 respectively (see also Table S1). Seven samples (from four early females, a late female and two early males) failed to amplify and were thus excluded in the analyses. Cq values, controlling for amplicon efficiency, were used to calculate the relative gene expression and standardized by a reference sample (Pfaffl, 2001).

### 2.6 Statistical analyses

#### 2.6.1 Within‐generational effects

We used linear models (LMs), linear mixed‐effect models (LMMs), generalized linear models (GLMs) and generalized linear mixed‐effect models (GLMMs) to test the effect of the photoperiod treatment on parental traits. All models included the experimental treatment (early or late) as a fixed factor and initial body mass (i.e., body mass prior to the experiment) as a covariate. The different models included different covariates, as appropriate (explained below). In all analyses, two‐way interactions between all fixed terms and the treatment were tested but excluded from the final models if statistically not significant, because their inclusion may produce inaccurate estimates for main effects (see Engqvist, 2005).

In females, the date of first spawning (in Julian day) was analyzed by using a LM. The number of spawning events was analyzed using a GLM with a Poisson distribution, including the date of first spawning as a covariate. Variation in female clutch size during the breeding season was analyzed with a GLMM with a Poisson error distribution, including the clutch order grouped in two classes, early clutches (i.e., the first 5 spawning events) and late clutches (i.e., 6–12 spawning events) and female identity as a random term.

In males, the effect of the experimental treatment on date of nest building was analyzed by using a LM. Relative size of the red area (measured twice) was first analyzed with a LMM including time (i.e., days) since the initiation of nest building and treatment as fixed effects and individual identity as a random effect. In addition, we reanalyzed red coloration by including the time of measurement (April and May) as a factor (instead of time since nest initiation) to test whether the treatment effect on sexual coloration differed according to sampling time.

Change in telomere length of breeders in dorsal fin tissue was first analyzed by a LMM, including the sex and sampling time (before and after the experiment) as fixed factors and individual identity as a random term. Additionally, to study the effects of the treatment on the relationship between reproductive effort and telomere length change or the abundance of *TERT* transcripts, we performed separate LM analyses by sex. Prior to the analyses, the within‐individual change in telomere length (telomere length in June—telomere length in February) was calculated and then corrected for regression to the mean (Verhulst et al., 2013). Thus, the average change is zero, and positive and negative values represent changes above and below the average change. In the LMs, we included initial telomere length (measured in February) and the number of spawning events (in the female model) or mean relative size of the red area (in the male model) as covariates.

#### 2.6.2 Transgenerational effects

The effect of the experimental treatment of the mother on egg size was analyzed using a LMM that included spawning date as a covariate and clutch identity nested within female identity as random effects. Hatching success (i.e., the proportion of eggs hatched per clutch) was analyzed by using a GLMM with a binomial distribution, including experimental treatments of parents, average egg size and spawning date as fixed effects and the identities of parents as random effects.

The transgenerational effects of the parental photoperiod treatment on offspring telomere length (at age 4 days) and growth (i.e., standard length and body mass at age 40 days) were analyzed by LMMs. In the models, the maternal and paternal photoperiod treatments, average egg size, spawning date and the telomere length of both parents (measured in June) in the offspring telomere length model were included as fixed effects and the identities of parents and clutch as random effects. Lastly, offspring survival (at the individual level) from hatching to age 40 days was analyzed with a Cox Proportional Hazard Model (CPHM). In the growth and survival models, the number of larvae in each family at hatching (initial group size) was also included as an additional fixed effect to account for any possible effect of density on development. Since the treatments of parents affected offspring mortality (see Results), we reanalyzed growth by replacing the initial group size with the group size at age 20 or 40 days in separate models.

Before the analyses of parental and offspring traits, first spawning date, nest building date, egg size, telomere length, and the expression of *TERT* gene were log‐transformed to improve data distribution and meet model assumptions of normality and homoscedasticity of residuals. The analyses were done by using the *lm*, *lmer* and *glmer* functions of the *lme4* package (Bates et al., 2014) and *Coxme* package (CPHM; Therneau & Therneau, 2015) in R v.3.5.1. The standardized coefficients and their confidence intervals (95% CI) were calculated as a measure of effect sizes in all models by using the *effectsize* package (Ben‐Shachar et al., 2020). Predicted slopes and partial residuals of the models were plotted by using *visreg* package (Breheny & Burchett, 2017). The significance of term was determined by *F* test in LMs, by *F* test with Satterthwaite approximation for degrees of freedom (using the *ANOVA* function of the *lmerTest* package) in LMMs (Kuznetsova et al., 2016), and by the Likelihood Ratio Test (LRT) in GLM, GLMMs and CPHM. The significant level was set at *p* = .05 and all statistical tests were two‐tailed. Unless specified, data are reported as estimated marginal means ± *SEM*.

## 3 RESULTS

### 3.1 Within‐generational effects on time of reproduction and other reproductive traits

**Figure 2:**
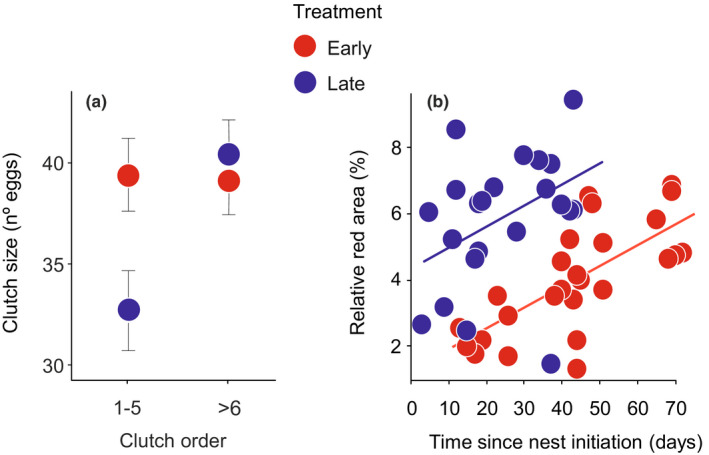
Effect of the photoperiod treatment on reproductive traits of (a) female and (b) male sticklebacks. (a) Clutch size (mean ± *SE*) in relation to treatment and clutch order of early (*N* = 15, red circles) and late (*N* = 10, blue circles) females. The clutch order was grouped in two classes, early clutches (i.e., the first 5 spawning events) and late clutches (i.e., 6–12 spawning events). (b) Relationship between size of the red area (proportion of body size) and time (days) since nest initiation in early (*N* = 13, red circles) and late (*N* = 16, blue circles) males sampled twice during the reproduction. Solid lines and circles are model predictions and partial residuals, respectively

In females, the experimental treatment of the breeding photoperiod influenced neither the date of first spawning event (LM: −0.02, 95% CI: −0.06, 0.03; *F*_1,22_ = 0.085, *p* = .774) nor the number of spawning events between March and June (GLM: −0.15, 95% CI: −0.49, 0.19; $\chi_{1}^{2}$ = 0.734, *p* = .392; see Table S2). However, the photoperiod treatment and clutch order class (i.e., early and late clutches) had an interacting effect on clutch size (GLMM: 0.16, 95% CI: 0.02, 0.30; $\chi_{1}^{2}$ = 5.116, *p* = .024; Table S2). In the early clutches (i.e., the first 5 spawning events), the late females laid smaller clutches than the early females, but this effect was not found in the last clutches (i.e., 6th–12th spawning events; Figure 2a).

As expected, the experimental treatment of breeding stimulation (via breeding photoperiod, nesting materials and presentation of a female) significantly affected the date of nest building in males (LM: 0.05, 95% CI: 0.03, 0.07; *F*_1,22_ = 24.388, *p* < .001). The late males completed nest building on average 22 days later than the early males (mean ± *SE*. date, 1 = 1 January: early males: 88.07 ± 3.62; late males: 109.75 ± 2.31). Relative area of red coloration increased with time since the initiation of nest building (Figure 2b; LMM: 0.46, 95% CI: 0.19, 0.74; *F*_1,34.71_ = 10.763, *p* = .002), and the late males showed a larger red area than the early males (late: 5.69 ± 0.55%; early: 4.04 ± 0.51%; 1.20, LMM: 95% CI: 0.54, 1.86; *F*_1,29.63_ = 12.823, *p* = .001). In the additional model including the time of measurement (April or May, two‐level factor) instead of time since the initiation of nest building, the effect of the treatment on red coloration was maintained (LMM: treatment: 0.67, 95% CI: 0.08, 1.26; *F*_1,22.68_ = 4.906, *p* = .037; time of measurement: 0.69, 95% CI: 0.28, 1.11; *F*_1,23.76_ = 10.681, *p* = .003), showing that the late males invested more in red coloration irrespective of the season.

### 3.2 Within‐generational effects on telomere length and *TERT* expression

Overall, telomere length in dorsal fin tissue was reduced on average by 13% between February and June (LMM: time: −1.06, 95% CI: −1.70, −0.42; *F*_1,51_ = 24.205, *p* < .001), and this reduction was not affected by the treatment or sex (LMM: treatment: −0.59, 95% CI: −1.27, 0.08; *F*_1,49_ = 0.022, *p* = .882; sex: 0.15, 95% CI: −0.54, 0.84; *F*_1,49_ = 0.262, *p* = .611; time × treatment: −1.42, 95% CI: −2.56, −0.28; *F*_1,49_ = 0.943, *p* = .336; time × sex: 0.52, 95% CI: −0.64, 1.69; F_1,49_ = 2.635, *p* = .111).

**Figure 3:**
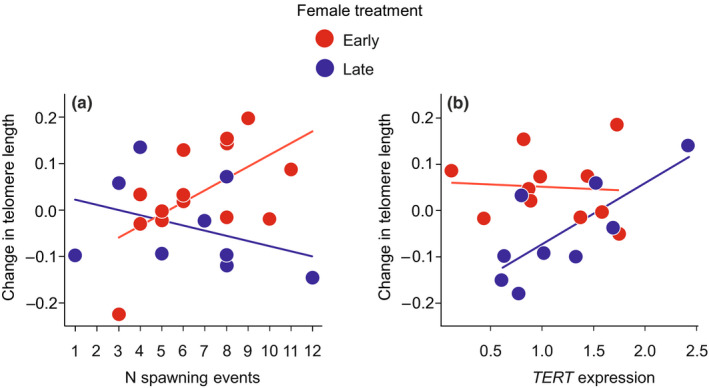
Relationships between corrected change in telomere length and (a) number of spawning events, and (b) the expression of TERT gene in female sticklebacks (early females, *N* = 14, red circles; late females, *N* = 9, blue circles). Change in telomere length between June and February was corrected for regression to the mean, and positive and negative values represent changes above and below the average change. Solid lines and circles are model predictions and partial residuals, respectively

In females, the change in telomere length was significantly affected by the interaction between reproductive effort, measured as the number of spawning events, and the photoperiod treatment (LM: treatment × number of spawning events: −1.62, 95% CI: −3.30, 0.07; *F*_1,18_ = 5.734, *p* = .028; Table S3). Thus, in the early females, change in telomere length increased with the number of spawning events but this relationship was negative in the late group (Figure 3a). In males, the change in telomere length was not affected by the photoperiod treatment, the size of the red area and their interaction (all *p* > .396; Table S3).

The abundance of *TERT* transcripts in muscle was not affected by the photoperiod treatment, the reproductive effort (i.e., the number of spawning events in females and the mean size of the red area in males) and their interaction in both sexes (all *p* > .125; Table S4). In females, however, the interaction between the treatment and the abundance of *TERT* transcripts significantly affected the change in telomere length (LM: 0.52, 95% CI: 0.05, 0.99; *F*_1,15_ = 5.597, *p* = .032; Table S5); late females with a lower abundance of *TERT* transcripts showed a higher level of telomere attrition, but this effect was not found in early females (Figure 3b). In males, neither the expression of *TERT* nor its interaction with the treatment affected the change in telomere length (both *p* > .576; Table S5).

### 3.3 Transgenerational effects on early development

Egg size of the late females tended to be smaller than that of the early females (late females = 1.612 ± 0.02 mm; early females = 1.655 ± 0.02), although this difference was not statistically significant (LMM: −0.08, 95% CI: −0.16, 0.01; *F*_1, 17.27_ = 3.530, *p* = .077). Egg size was not affected by the spawning date and its interaction with the experimental treatment of female breeders (both *p* > .529). Hatching success was not affected by the treatments of parents (GLMM: father treatment: 0.07, 95% CI: −0.15, 0.29; $\chi_{1}^{2}$ = 1.521, *p* = .217; mother treatment: 0.08, 95% CI: −0.17, 0.32; $\chi_{1}^{2}$ = 1.329, *p* = .249) and their interaction (GLMM: 0.10, 95% CI: −0.26, 0.46; $\chi_{1}^{2}$ = 0.323, *p* = .569).

### 3.4 Transgenerational effects on telomere length of larvae

**Figure 4:**
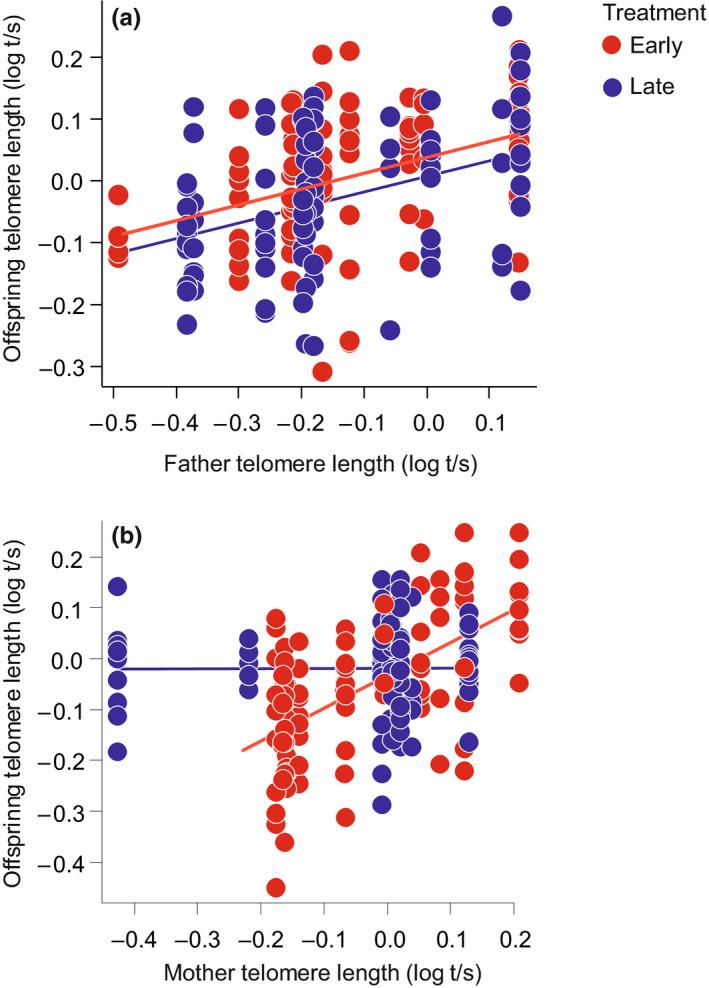
Telomere length relationships between parents (dorsal fin tissue sampled in June) and offspring (larvae sampled at age 4 days, *N* = 189). (a) Father–offspring relationships in F1 families obtained from the early (red circles) and late (blue circles) fathers. (b) Mother–offspring relationships in F1 families obtained from the early (red circles) and late (blue circles) mothers. Solid lines and circles are model predictions and partial residuals, respectively

Telomere length of larvae sampled at age 4 days was not affected by the parental treatments and their interaction (all *p* > .239, Table S6). Interestingly, offspring telomere length was positively related to paternal telomere length (LMM: 0.29, 95% CI: 0.10, 0.48; *F*_1, 14.2_ = 9.128, *p* = .009), irrespective of the paternal treatment (Figure 4a). There also was a positive correlation between offspring and maternal telomere length but only in the families obtained from the mothers treated with an early breeding photoperiod (LMM: mother telomere length × mother treatment: −0.58, 95% CI: −1.04, −0.12; *F*_1, 10.9_ = 6.201, *p* = .030; Figure 4b). Additionally, larvae from clutches with a larger mean egg size had longer telomeres (LMM: 0.27, 95% CI: 0.05, 0.49; *F*_1, 29.9_ = 5.683, *p* = .024; Table S6).

### 3.5 Transgenerational effects on growth and survival

**Figure 5:**
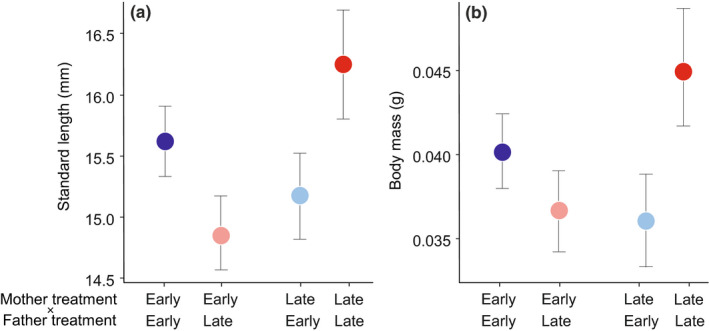
(a) Offspring standard length and (b) body mass at age 40 days in relation to the photoperiod treatment of their parents. Data are estimated marginal mean ± *SEM*

Initial group size (i.e., number of larvae in the family at hatching) negatively affected the growth in body mass and length (Table S7). There was a significant interacting effect of the photoperiod treatment experienced by the two parents on standard length and body mass of juveniles at age 40 days (LMM: standard length: 1.00, 95% CI: 0.26, 1.73; *F*_1, 29.25_ = 7.391, *p* = .011; body mass: 0.95, 95% CI: 0.23, 1.67; *F*_1,29.31_ = 6.852, *p* = .014; Table S7). Juveniles grew the fastest when both parents experienced a late breeding photoperiod and the slowest when the photoperiod treatment of parents mismatched, that is, early mother × late father and late mother × early father (Figure 5). Similar results were achieved when the group size at 20 or 40 days was included as a covariate in the analyses (Table S8).

**Figure 6:**
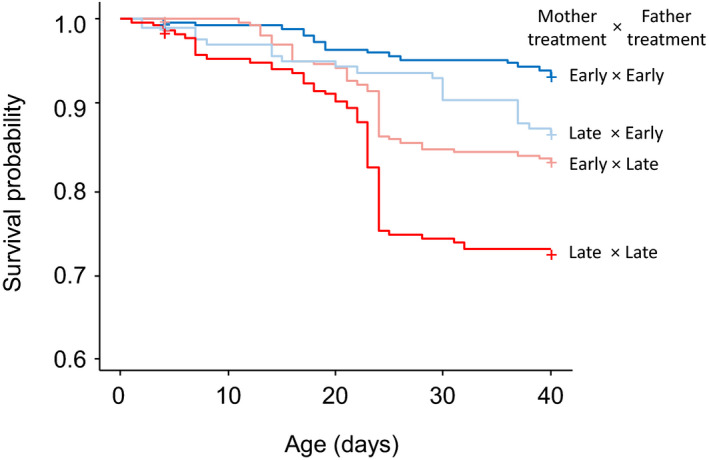
Estimated survival functions of juvenile sticklebacks from parents with different photoperiod treatments (late mother × early father, *N* = 199; early mother × late father, *N* = 314; both late parents, *N* = 284; both early parents, *N* = 295). Crosses mark censorship events, that is, individuals sampled at age 4 days and individuals who were still alive at the end of the study (at age 40 days)

Analysis of survival until age 40 days revealed that interaction between the father and mother photoperiod treatments significantly affected offspring juvenile survival (CPHM: father × mother treatment: 1.00, 95% CI: 0.26, 1.73; $\chi_{1}^{2}$ = 4.189, *p* = .041); offspring survival was the highest when both parents came from the early group (survival rate 93%), the lowest when both parents came from the late group (72%) and showed intermediate values when parents came from different experimental treatments (i.e., early mother × late father: 85%; late mother × early father: 89%; Figure 6).

## 4 DISCUSSION

Our results demonstrate that changes in photoperiod during the prebreeding period induced sex‐specific effects on the breeders' reproductive strategy, with carry‐over consequences on offspring life histories and telomere length. In response to a delay in the onset of reproductive photoperiod, females reduced their investment in egg production, especially in the early clutches, but males increased their investment in sexual signalling. Our results of telomere dynamics indicate that the late females probably paid a higher cost of egg production (in terms of number of spawning events) than did the early females through an acceleration in telomere shortening not observed for the early females. However, there was no such somatic penalty, in terms of telomere loss, of an increased investment in sexual signalling for both early and late males. Irrespectively of these sex‐specific effects on the within‐generation performances, the photoperiod treatment of the two parents similarly affected the growth and survival of their offspring. These results suggest that similar transgenerational effects of the environment experienced by mothers and fathers may arise by different mechanisms.

Our results show that the experimental manipulation of reproductive photoperiod did not alter the onset of spawning, but the late females laid smaller clutches than the early females during the early season. In sticklebacks, vitellogenesis, the deposition of yolk into oocytes, is stimulated by changes in photoperiod just before the beginning of the spawning season (Baggerman, 1980; Ishikawa & Kitano, 2020). Because the late females matured at the same time as the early females but started receiving a long‐day photoperiod later in the season, they probably had less time to accumulate enough energetic resources for egg production in the early breeding season (Elkin & Reid, 2005; Garrido et al., 2007; Glazier, 1999). The late females increased their productivity later in the season and showed a comparable clutch size with the early females, but increasing reproductive effort probably incurred a somatic cost to the late females. In the early females, telomere length increased during the breeding season, especially in more fecund females, suggesting that high‐quality females were able to invest heavily in both reproduction and telomere maintenance/growth (see Hamel et al., 2009; Sudyka, 2019). However, the relationship between change in telomere length and the number of spawning events was negative in the late females. This suggests that the trade‐off between reproductive effort and telomere growth and maintenance (Chatelain et al., 2020; Gao & Munch, 2015) became apparent in the late females due to their relatively poor preparation for reproduction. Interestingly, the change in telomere length was positively associated with the abundance of *TERT* transcripts in the late, but not in the early, females. Thus, the cost of reproduction in the late females, in terms of telomere shortening, may be related to their capacity to restore their telomeres via telomerase enzyme, highlighting the potential role of telomerase activity in telomere dynamics and life‐history trade‐offs in ectotherms (Hatakeyama et al., 2016; Olsson et al., 2018).

In contrast to the response by females, the late males showed a relatively high reproductive investment in red nuptial coloration in comparison to the early males throughout the season. In the three‐spined stickleback, adult females, but not males, maintain a large investment in somatic growth throughout the breeding season (Bell & Foster, 1994; Ostlund‐Nilsson et al., 2006). Thus, it is likely that sticklebacks experience sex‐specific trade‐offs between growth and reproductive activities during the preparation stage as well as during the reproduction. Indeed, male sticklebacks start preparing for reproduction earlier than females, and this probably induces sex differences in morphology, behavior, and metabolism at the juvenile stage (Velando et al., 2017). Males store carotenoids in various tissues during their development to be later mobilized and deposited into the integument during the breeding season (Black et al., 2014). Thus, it is likely that the late males had more time than the early males to accumulate carotenoids before the onset of the breeding season (i.e., before investing in other costly reproductive activities such as nest building and courtship) and so to increase their expression of red nuptial coloration when finally exposed to a long‐day photoperiod. It is also possible that the late males strategically increased their investment in nuptial coloration (e.g., Kim & Velando, 2014) when the breeding photoperiod was postponed and thus their opportunities for reproduction were reduced in this seasonally breeding population (Candolin, 2000; Lindström et al., 2009). Importantly, although carotenoids are involved in important physiological processes for somatic maintenance, such as antioxidant protection (Pike et al., 2007), the increase of carotenoid‐based coloration did not affect telomere loss in the late males. Our results show that the environment experienced by male and female breeders before their reproduction can have different effects on telomere dynamics (see also Noreikiene et al., 2017). However, it is also possible that we could not detect the interacting effect of the treatment and reproductive effort on telomere dynamics of males, because the males were not allowed to perform costly parental care (i.e., fanning behavior for aerating eggs in the nest) in our experiment.

Notably, we found clear evidence that environmentally induced changes in parental reproductive strategies affected offspring growth and survival. Growth differences remained significant when controlling for the number of fish per tank during the study period, suggesting that these differences cannot be attributed to changes in sibling competition. Offspring growth rate was the fastest when both parents received a long‐day photoperiod later in the season and the slowest when the photoperiod treatment mismatched between the two parents. Offspring from the early mothers and fathers showed an intermediate growth rate, and interestingly, had the highest survival rate, suggesting the optimal offspring performances of this group. In this species, accelerated growth increases oxidative damage (Kim et al., 2019) and shorten lifespan (Ab Ghani & Merilä, 2014; Inness & Metcalfe, 2008). Despite these fitness costs, fast growth may still be adaptive for the juveniles from the late parents if, for example, it accelerates their maturation and improves their opportunities for reproduction (Berglund, 1995; Rowe & Thorpe, 1990) in the same environments with reduced time to prepare for reproduction as those experienced by their parents. Information about the environments experienced by the parents may be transmitted to offspring by gametes (Taborsky, 2017). Interestingly, offspring obtained from the mismatched crosses between pairs with different photoperiod treatments showed relatively slow growth and low survival. A possible reason is that the mismatch in information transmitted from the two parents through genetic cues and quality (McNamara et al., 2016; Monaghan & Metcalfe, 2019) perhaps incurred a fitness cost to the offspring due to its contrasting influence in developmental pathways (Meunier & Kölliker, 2012). Since the eggs were artificially incubated under standardized conditions, the transgenerational effects of the environment experienced by parents are attributed to only changes in parental gametes.

In this study, we explored telomere length of parents and offspring as a potential mechanism by which the environment of parents determines life‐history traits of offspring (Monaghan, 2010). Telomere length of fathers and offspring were highly correlated, but we found no evidence that paternal treatment affected offspring telomere length. Thus, the mechanism underlying the transgenerational effects of changes in male reproductive strategies on offspring survival reported here was not related to telomeres. It is possible that changes in male germline quality and integrity due to the increased investment of the late males in their nuptial coloration gave rise to the reduced offspring survival. Indeed, our previous evidence indicates that, in male sticklebacks, sexual coloration trades off against sperm DNA integrity (Kim et al., 2019) and offspring viability (Kim & Velando, 2016). Maternal treatment did not have a clear effect on offspring telomere length, but the somatic telomere length resemblance between mothers and offspring was obscured in the late photoperiod treatment. Since we did not measure telomere length in mothers' germline, the mechanisms that cause this disruption are difficult to elucidate. However, it is likely that the negative interacting effects of the photoperiod treatment and reproductive investment on telomere dynamics of females (see above) disrupted their genetic effects on offspring telomere length. Interestingly, telomere length of the 4‐day‐old larvae was positively correlated with egg size, suggesting the important role of maternal nutrients in preventing telomere shorting during the embryonic development (McLennan et al., 2018). Thus, it is also possible that changes in maternal effects in response to the experimental photoperiod resulted in the differential mother–offspring relationships in telomere length between the early and late photoperiod treatments.

Our study experimentally demonstrates that changes in environmental cues experienced prior to reproduction alter the reproductive strategies of seasonal breeders and that these effects last more than one generation. Our results highlight that different responses of male and female breeders to changes in environmental cues determine growth and survival of offspring. We show that the mechanisms underlying the transgenerational effects may include both genetic and nongenetic effects of parental conditions but not telomere length. The experimental demonstration of the transgenerational effects of changes in reproductive strategies provides interesting insights into the long‐lasting influences of environmental changes on the development of life‐history phenotypes in seasonal animal populations.

## CONFLICT OF INTEREST

The authors declare no competing interests.

## AUTHOR CONTRIBUTIONS

**Náyade Álvarez‐Quintero:** Formal analysis (equal); investigation (equal); methodology (equal); writing–original draft (equal). **Alberto Velando:** Formal analysis (equal); funding acquisition (lead); investigation (equal); project administration (lead); supervision (equal); writing–original draft (equal). **Jose C. Noguera:** Investigation (equal); supervision (equal); writing–original draft (equal). **Sin‐Yeon Kim:** Funding acquisition (lead); investigation (equal); methodology (equal); project administration (lead); supervision (equal); writing–original draft (equal).

## ETHICAL APPROVAL

This experiment was approved by the Animal Experiment Ethics Committee of the Universidade de Vigo and the Xunta de Galicia (ES360570181401/17/FUN01/BIOL AN.08/SYK01).

## Supporting information

Supplementary Material

## Data Availability

All data needed to evaluate the conclusions in this paper and/or in the Supporting Information. The raw data can also be found in the Figshare digital repository (https://doi.org/10.6084/m9.figshare.12764246).
