# Supplementary material for: Environment‐induced changes in reproductive strategies and their transgenerational effects in the three‐spined stickleback
Source: Ecol Evol. 2020 Dec 9;11(2):771–83. doi: 10.1002/ece3.7052 (PMC7820166; doi:10.1002/ece3.7052)
Supplement: Supplementary file 1 — Supplementary Material [file ECE3-11-771-s001.pdf]

SUPPORTING INFORMATION (SI)

Environment-induced changes in reproductive strategies and their transgenerational effects in the three-spined stickleback

SUPPORTING FIGURES

Figure S1. Relationship between clutch size (i.e. number of eggs) and clutch order in individual females in the early (15 females and 76 clutches, red circles and lines) and late (10 females and 44 clutches, blue circles and lines) groups. The figure also shows mean ± *S.E.* of clutch size and clutch order, and their distributions in the early (red shadow) and late groups (blue shadow).

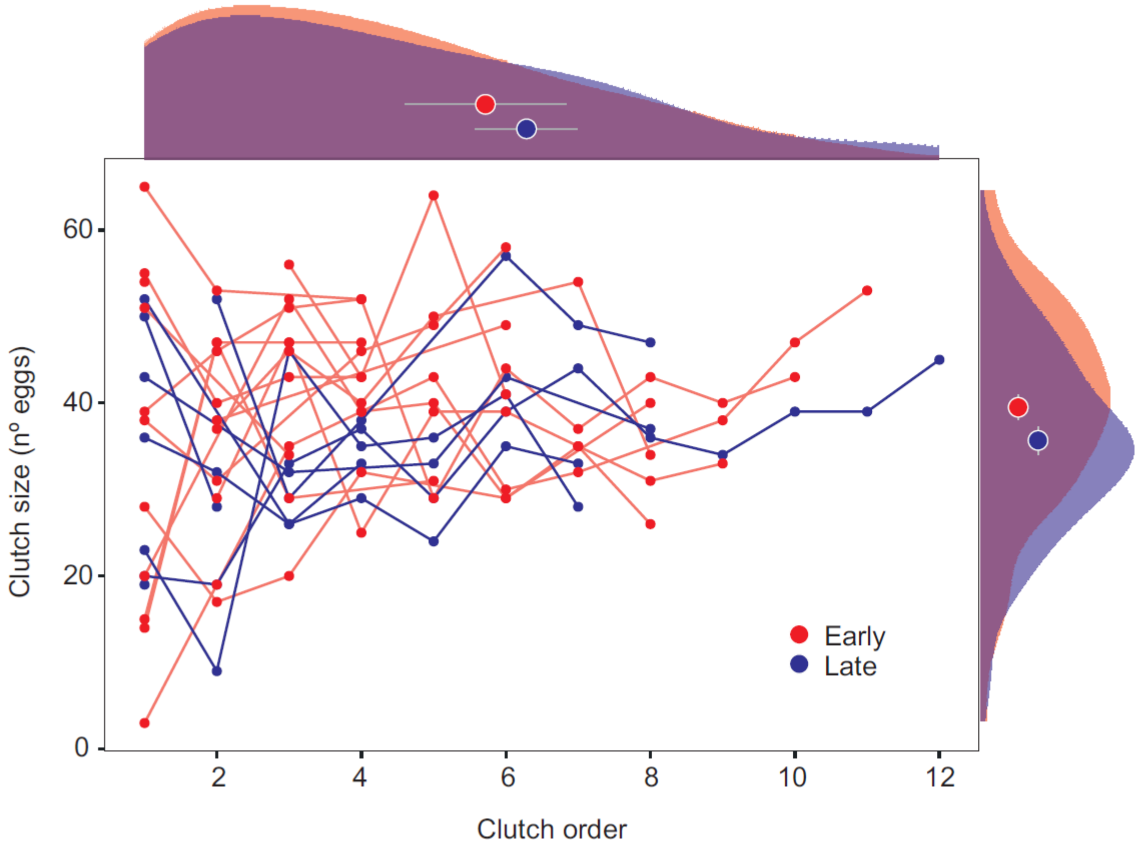

Figure S2. (a) Offspring standard length and (b) body mass at age 40 days in relation to the photoperiod treatment of their parents with visible data points. Data are estimated marginal mean ± *S.E.M.*

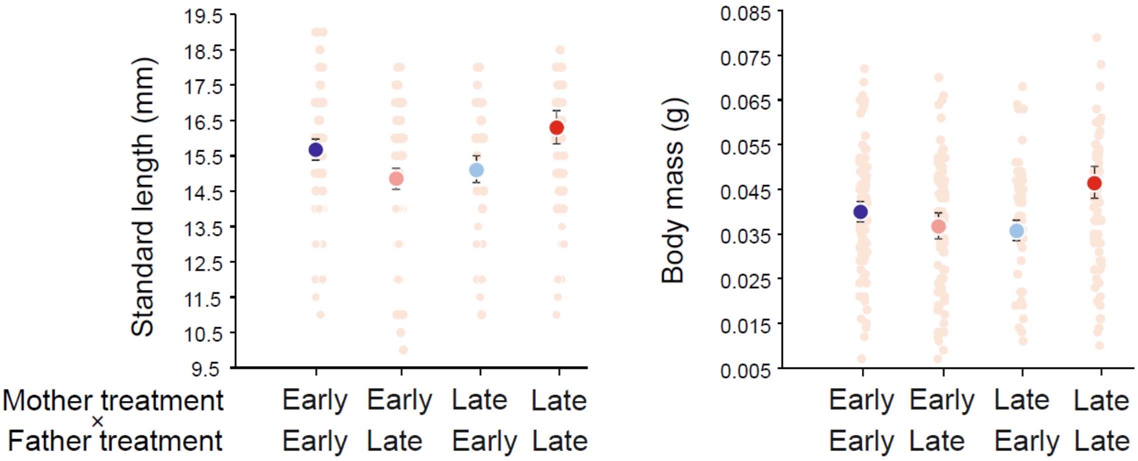

SUPPORTING TABLES

Table S1. Primers used for real-time qPCR.

Gene	Ensembl gene ID	Sequence (5'-3') Forward (F) Reverse (R)	Tm (ºC, F/R)	Efficiency	Intra/inter assay CV
TEL	ENSGACT000000	F: CGGTTTGTTTGGGTTTGGGTTTGG GTTTGGGTTTGGGTT R: GGCTTGCCTTACCCTTACCCTTAC CCTTACCCTTACCCT	84.9/78.6	1.861	0.79/0.37
TERT	ENSGACT000000	F: GCCCAGAGTTTACCTTACGG R:GAGAATCATTCCTTTGTTGCTGAG	62.4/64.9	1.924	0.30/0.06
GAPDH	ENSGACT000000	F: GAGACGTGACCATTGAGGGG R: TGTGCGGTGGGCTTTATGAT	66.9/67.4	1.878	0.14/0.15
EF1α	ENSGACT00000002834	F: CTTCTCCAACTATCCTCCCCTC R: GAGAAGAGGATCCAGGGTAAGG	65.6/66.4	1.859	0.31/0.02

Table S2. Summary of the linear model (LM), generalized linear model (GLM) or generalized linear mixed-effect model (GLMM) of female reproductive traits: first spawning date, number of spawning events and clutch size. Statistically not significant interaction was removed from the presented models of first spawning date (treatment × body mass: $F_{1,\ 21}$= 1.316, p = 0.264), number of spawning events (treatment × first spawning event: = 2.647, p = 0.104; treatment × body mass: χ^2_1 = 0.007, p = 0.934) and clutch size (treatment × body mass: χ^2_1 = 1.038, p = 0.308). Significant p-values are highlighted in bold.

	First spawning date				N of spawning events		
Variable	Estimate ± SE	df	F	p	Estimate ± SE	χ^2_1	p
Intercept	4.85 ± 0.15				4.25 ± 1.73		
Treatment (late)	-0.06 ± 0.10	1, 22	0.085	0.774	-0.15 ± 0.17	0.734	0.392
Female body mass	-0.49 ± 0.22	1, 22	5.052	**0.035**	-0.52 ± 0.40	1.725	0.189
First spawning date	-	-	-	-	-0.46 ± 0.35	1.697	0.193

	Clutch size		
Variable	Estimate ± SE	χ^2_1	p
Intercept	3.44 ± 0.12		
Treatment (late)	-0.14 ± 0.08	1.485	0.223
Female body mass	0.39 ± 0.17	5.041	0.025
Clutch order (late)	0.07 ± 0.04	13.696	**<0.001**
Treatment (late) × Clutch order (late)	0.16 ± 0.07	5.116	**0.024**
Random effect	Variance	X^2_1	p
Female identity	0.02	65.488	**<0.001**

Table S3. Summary of the LM analyses of change in telomere length according to individual reproductive investment in F0 male and female sticklebacks. In the analysis of males, statistically not significant interaction was removed from the presented model (treatment × size of the red area: $F_{1,\ 20}$ = 0.203, p = 0.657). Significant p-values are highlighted in bold.

	Change in telomere length (females)				Change in telomere length (males)			
Variable	Estimate ± SE	df	F	p	Estimate ± SE	df	F	p
Intercept	-0.14 ± 0.08				-0.04 ± 0.06			
Treatment (Late)	0.17 ± 0.11	1,18	3.666	0.071	-0.01 ± 0.05	1,21	0.119	0.734
Initial telomere length	-0.15 ± 0.08	1,18	4.342	0.052	-0.19 ± 0.9	1,21	4.179	0.054
N of spawning events (NSE)	0.02 ± 0.01	1,18	0.751	0.397	-	-	-	-
Size of the red area (mean)	-	-	-	-	0.01 ± 0.01	1,21	0.750	0.396
Treatment × NSE	-0.04 ± 0.01	1,18	5.734	**0.028**	-	-	-	-

Table S4. Summary of the LM analyses of *TERT* expression according to individual reproductive investment in F0 male and female sticklebacks. Statistically not significant interaction was removed from the presented models (females, treatment × number of spawning events: $F_{1, 15}$ = 0.067, p = 0.799; males, treatment × size of the red area: $F_{1, 18}$ = 0.103, p = 0.751).

	TERT expression (females)				*TERT* expression (males)			
Variable	Estimate ± SE	df	*F*	*p*	Estimate ± SE	df	*F*	*p*
Intercept	0.05 ± 0.43				-0.04 ± 0.06			
Treatment (late)	0.14 ± 0.30	1,16	0.391	0.540	-0.02 ± 0.05	1, 19	0.036	0.851
Initial telomere length	-0.13 ± 0.60	1,16	0.045	0.835	-0.19 ± 0.09	1, 19	0.967	0.338
N of spawning events	-0.09 ± 0.05	1,16	2.624	0.125	-	-	-	-
Size of the red area	-	-	-	-	0.01 ± 0.01	1, 19	2.093	0.164

Table S5. Summary of the LM analyses of change in telomere length in F0 male and female sticklebacks, including the expression of TERT gene as a covariate. Statistically not significant interaction was removed from the presented model (males, treatment × *TERT* expression: $F_{1, 22}$ = 0.144, p = 0.708). Significant *p*-values are highlighted in bold.

	Change in telomere length including *TERT* (males)				Change in telomere length including *TERT* (females)			
Variable	Estimate ± SE	df	*F*	*p*	Estimate ± SE	df	*F*	*p*
Intercept	0.03 ± 0.04				0.05 ± 0.02			
Treatment (late)	-0.03 ± 0.04	1, 23	0.119	0.734	-0.11 ± 0.04	1, 15	7.535	**0.015**
Initial telomere length	-0.26 ± 0.10	1, 23	7.478	**0.012**	-0.02 ± 0.08	1, 15	0.986	0.336
TERT expression	0.04 ± 0.04	1, 23	0.321	0.576	-0.01 ± 0.03	1, 15	1.151	0.300
Treatment × *TERT* expression	-	-	-	-	0.17 ± 0.07	1, 15	5.597	**0.032**

Table S6. Summary of the GLMM or LMM analyses of hatching success and offspring telomere length. Statistically not significant interaction, mother treatment × father treatment, was removed from the presented models (hatching success: χ^2_1 = 0.322, p = 0.570; offspring telomere length: $F_{1, 8.69}$ = 1.595, p = 0.239). Significant *p*-values are highlighted in bold.

	Hatching success			Telomere length			
Variable	Estimate ± SE	χ^2_1	*p*	Estimate ± SE	df	*F*	*p*
Intercept	-0.94 ± 3.10			-0.38 ± 0.28			
Mother treatment (late)	0.12 ± 0.10	1.329	0.249	0.01 ± 0.04	1, 10.83	0.121	0.735
Father treatment (late)	0.11 ± 0.09	1.521	0.217	-0.03 ± 0.02	1, 8.67	1.375	0.272
Clutch date	-0.04 ± 0.59	0.004	0.949	-0.00 ± 0.00	1, 19.18	0.003	0.953
Egg size (mean)	1.75 ± 1.06	2.728	0.098	0.87 ± 0.36	1, 29.96	5.683	**0.024**
Paternal telomere length	-	-	-	0.26 ± 0.08	1, 14.22	9.128	**0.009**
Maternal telomere length	-	-	-	0.64 ± 0.19	1, 11.59	6.225	**0.029**
Maternal telomere length × Mother treatment (late)	-	-	-	-0.64 ±-0.26	1, 10.86	6.201	**0.030**
Random effects	Variance		*p*	Variance		χ^2_1	*P*
Mother identity	0[a]	-	1	4.37E-3		0.633	0.426
Father identity	0[a]	-	1	8.56E-4		1.460	0.227
Clutch identity	0[a]	-	1	1.426E-3		0.252	0.615

[a] Parameter estimate bound at zero; hence no *X* was estimated.

Table S7. Summary of the LMM analyses of offspring growth (i.e. standard length and body mass), including number of fry per family at hatching as a covariate. Statistically not significant interactions were removed from the models, and significant *p*-values are highlighted in bold.

	Standard length				Body mass			
Variable	Estimate ± SE	df	*F*	*p*	Estimate ± SE	df	*F*	*p*
Intercept	3.35 ± 11.45				0.07 ± 0.09			
Mother treatment (late)	-0.47 ± 0.45	1, 28.96	1.666	0.207	-0.00 ± 0.00	1, 28.99	0.968	0.333
Father treatment (late)	-0.74 ± 0.39	1, 27.74	0.378	0.544	-0.00 ± 0.00	1, 27.81	2.232	0.146
Mother × Father Treatment (late × late)	1.88 ± 0.69	1, 29.25	7.391	**0.011**	0.01 ± 0.00	1, 29.31	6.852	**0.014**
Clutch date	2.45 ± 2.19	1, 32.54	1.249	0.272	-0.00 ± 0.02	1, 32.53	0.041	0.840
Egg size (mean)	6.30 ± 3.82	1, 33.74	2.725	0.108	0.03 ± 0.03	1, 33.66	0.882	0.354
N of fry per family at age 0 days	-0.11 ± 0.02	1, 36.41	33.284	**< 0.001**	-0.00 ± 0.00	1, 36.27	43.005	**< 0.001**
Random effects	Variance		χ^2_1	*p*	Variance		χ^2_1	*p*
Mother identity	0[a]		-	1	0[a]		-	1
Father identity	0[a]		-	1	0[a]		-	1
Clutch identity	0.479		2.108	0.146	2.889e-5		2.518	0.112

[a] Parameter estimate bound at zero; hence no *X* was estimated.

Table S8. Summary of the LMM analyses of offspring growth (i.e. standard length and body mass), including (a) number of larvae in the family at age 20 days or (b) number of larvae in the family at age 40 days. Statistically not significant interactions were removed from the presented models, and significant *p*-values are highlighted in bold.

(a)

	Growth (standard length)				Growth (body mass)			
Variable	Estimate ± SE	df	*F*	*p*	Estimate ± SE	df	*F*	*p*
Intercept	1.46 ± 11.65				0.06 ± 0.09			
Mother treatment (Late)	-0.47 ± 0.43	1, 18.82	1.606	0.220	-0.00 ± 0.00	1, 20.33	0.837	0.371
Father treatment (Late)	-0.83 ± 0.40	1, 12.74	0.070	0.795	-0.00 ± 0.00	1, 13.68	0.937	0.350
Mother × Father Treatment (Late × Late)	1.84 ± 0.67	1, 19.26	7.430	**0.013**	0.01 ± 0.00	1, 21.01	6.94	**0.015**
Clutch date	2.69 ± 2.23	1, 30.77	1.461	0.236	-0.00 ± 0.02	1, 31.19	0.024	0.878
Egg size (mean)	7.56 ± 3.88	1, 31.36	3.800	0.060	0.04 ± 0.03	1, 32.18	1.629	0.211
N of fry per family at age 20 days	-0.11 ± 0.02	1, 35.67	28.862	**< 0.001**	-0.00 ± 0.00	1, 35.37	35.375	**< 0.001**
Random effects	Variance		χ^2_1	*p*	Variance		χ^2_1	*p*
Mother identity	0[a]		-	1	0[a]		-	1
Father identity	0.106		-	1	1.082e-5		-	1
Clutch identity	0.388		1.480	0.224	1.975e-5		1.296	0.255

(b)

	Standard length				Body mass			
Variable	Estimate ± SE	df	*F*	*p*	Estimate ± SE	df	*F*	*p*
Intercept	-4.38 ± 12.38				0.01± 0.09			
Mother treatment (late)	-0.53 ± 0.43	1, 18.71	1.636	0.216	-0.00 ± 0.00	1, 19.40	0.772	0.390
Father treatment (late)	-0.94 ± 0.43	1, 13.56	0.024	0.879	-0.00 ± 0.00	1, 14.54	0.547	0.471
Mother × Father Treatment (late × late)	1.99 ± 0.70	1, 19.72	8.118	**0.010**	0.01 ± 0.00	1, 20.38	7.378	**0.013**
Clutch date	3.86 ± 2.37	1, 30.83	2.636	0.115	-0.00 ± 0.02	1, 29.24	0.109	0.743
Egg size (mean)	7.39 ± 4.09	1, 33.03	3.263	0.080	0.03 ± 0.03	1, 32.00	1.225	0.276
N of larvae at age 40 days	-0.10 ± 0.02	1, 38.79	21.404	**< 0.001**	-0.00 ± 0.00	1, 37.05	26.014	**< 0.001**
Random effects	Variance		χ^2_1	*p*	Variance		χ^2_1	*P*
Mother identity	7.033e-13		-	1	3.214e-17		-	1
Father identity	0.261		-	1	2.023e-5		-	1
Clutch identity	0.352		1.521	0.217	1.868e-5		1.061	0.303

[a] Parameter estimate bound at zero; hence no *X* was estimated.

Table S9. Group size (mean±SE) in the growth tanks at age 1, 20 and 40 days according to experimental treatments.

		Age		
Mother treatment	Father treatment	1 day	20 days	40 days
early	early	24.4 ± 2.3	23.5 ± 2.4	22.7 ± 2.2
early	late	23.4 ± 2.1	22.0 ± 1.9	19.5 ± 2.4
late	early	15.0 ± 3.9	14.1 ± 3.8	12.9 ± 3.8
late	late	26.5 ± 3.7	20.3 ± 4.1	19.2 ± 3.9
